# Acetylation promotes BCAT2 degradation to suppress BCAA catabolism and pancreatic cancer growth

**DOI:** 10.1038/s41392-020-0168-0

**Published:** 2020-05-29

**Authors:** Ming-Zhu Lei, Xu-Xu Li, Ye Zhang, Jin-Tao Li, Fan Zhang, Yi-Ping Wang, Miao Yin, Jia Qu, Qun-Ying Lei

**Affiliations:** 1 Department of Biochemistry and Molecular Biology, School of Basic Medical Sciences, Shanghai Medical College, Fudan University, 131 Dong’an Road, Shanghai, 200032 China; 2 Cancer Metabolism Laboratory and the Shanghai Key Laboratory of Medical Epigenetics, the International Co-laboratory of Medical Epigenetics and Metabolism, Ministry of Science and Technology, Institutes of Biomedical Sciences, Shanghai Medical College, Fudan University, 131 Dong’an Road, Shanghai, 200032 China; 3 Cancer Institute, Fudan University Shanghai Cancer Center and Department of Oncology, Shanghai Medical College, Fudan University, 270 Dong’an Road, Shanghai, 200032 China; 4 State Key Laboratory of Medical Neurobiology, Fudan University, 131 Dong’an Road, Shanghai, 200032 China

**Keywords:** Paediatric cancer, Cancer metabolism, Biochemistry

## Abstract

Pancreatic ductal adenocarcinoma (PDAC) is well-known for inefficient early diagnosis, with most patients diagnosed at advanced stages. Increasing evidence indicates that elevated plasma levels of branched-chain amino acids (BCAAs) are associated with an increased risk of pancreatic cancer. Branched-chain amino acid transaminase 2 (BCAT2) is an important enzyme in BCAA catabolism that reversibly catalyzes the initial step of BCAA degradation to branched-chain acyl-CoA. Here, we show that BCAT2 is acetylated at lysine 44 (K44), an evolutionarily conserved residue. BCAT2 acetylation leads to its degradation through the ubiquitin–proteasome pathway and is stimulated in response to BCAA deprivation. cAMP-responsive element-binding (CREB)-binding protein (CBP) and SIRT4 are the acetyltransferase and deacetylase for BCAT2, respectively. CBP and SIRT4 bind to BCAT2 and control the K44 acetylation level in response to BCAA availability. More importantly, the K44R mutant promotes BCAA catabolism, cell proliferation, and pancreatic tumor growth. Collectively, the data from our study reveal a previously unknown regulatory mechanism of BCAT2 in PDAC and provide a potential therapeutic target for PDAC treatment.

## Introduction

**Fig. 1:**
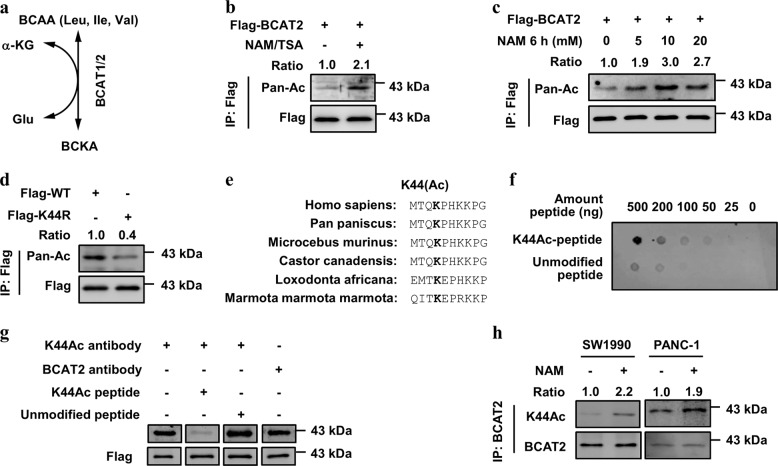
BCAT2 is acetylated mainly at lysine 44. **a** Diagram of the BCAA catabolic pathway. As shown, branched-chain amino acids (leucine, isoleucine, and valine) are reversibly transaminated by branched-chain amino acid transaminase 1/2 to produce BCKA. **b** Exogenous BCAT2 is acetylated. Flag-BCAT2 WT was ectopically expressed in HEK293T cells and treated with the deacetylase inhibitors NAM (5 mM, 6 h) and TSA (10 μM, 16 h). BCAT2 acetylation was detected with an anti-acetyl lysine (Pan-Ac) antibody by western blotting. The relative BCAT2 acetylation level was normalized to that of Flag-BCAT2 protein. **c** NAM treatment increases BCAT2 acetylation in a dose-dependent manner. Flag-BCAT2 was transfected into HEK293T cells. Cells were treated with NAM at the indicated concentrations for 6 h. The relative BCAT2 acetylation level was normalized to that of the Flag-BCAT2 protein. **d** The K44R mutant has decreased BCAT2 acetylation. Flag-BCAT2 WT and the K44R mutant were ectopically expressed in HEK293T cells, and BCAT2 acetylation was measured by western blotting. The relative BCAT2 acetylation level was normalized to that of the Flag-BCAT2 protein. **e** K44 is conserved and located in the N-terminus. The sequences around BCAT2 K44 from different species were aligned. **f** The K44 site-specific acetylation antibody can detect the acetylated peptide, but not detect an unmodified peptide. The different amounts of either acetylated K44 peptide or unmodified peptide were spotted onto a nitrocellulose membrane, as indicated, and probed with the anti-Ac BCAT2 (K44) antibody (K44Ac). **g** The K44Ac peptide, but not the unmodified peptide, blocks the K44 site-specific antibody. The K44Ac antibody was incubated with K44Ac peptide or an unmodified peptide for 3 h at 4 °C and used in western blotting. The BCAT2 antibody was included as a control. **h** NAM increases endogenous BCAT2 acetylation. SW1990 and PANC-1 cells were treated with NAM for the indicated times. Endogenous BCAT2 protein was purified and detected with K44Ac antibody. The relative BCAT2 K44 acetylation level was normalized to that of BCAT2 protein. Data are representative of three independent experiments in **b**, **c**, **d**, **f**, **g**, **h**

BCAAs (leucine, isoleucine, and valine) are the most hydrophobic and essential amino acids for protein synthesis and molecular signals.^1^ Circulating levels of BCAAs are tightly regulated. The pathway of BCAA catabolism is shown in Fig. 1a. All three BCAAs are reversibly transaminated by branched-chain amino acid transaminase 1/2 (BCAT1/2) to form branched-chain α-keto acid (BCKA), and then, BCKA is oxidatively decarboxylated by the branched-chain keto-acid dehydrogenase (BCKDH) complex. The final metabolites enter the TCA cycle for energy production. In humans, *BCAT1* encodes a cytoplasmic protein that is primarily expressed in the brain, while *BCAT2* encodes a mitochondrial protein that is ubiquitously expressed in all organs (except hepatocytes).^2^ BCAT2 reversibly catalyzes the initial step of BCAA catabolism to produce BCKA and glutamate.

Abnormal BCAA metabolism is associated with obesity, insulin resistance, type 2 diabetes, heart disease, and cancer.^3–6^ BCAAs are important nitrogen sources and carbon sources for tumor growth. Tumor cells obtain BCAAs from either the circulation or surrounding tissue. Increasing evidence indicates that elevated levels of BCAAs in plasma are positively associated with pancreatic cancer risk.^7,8^

Recent studies have largely focused on BCAT1 in several cancer contexts.^9–11^ However, few reports have addressed BCAT2 in tumors, which has an unclear function in pancreatic ductal adenocarcinoma (PDAC).^12,13^ Although studies have found that the mRNA level of *BCAT2* is regulated by Kruppel-like factor 15 (KLF15) and sterol regulatory element-binding protein 1 (SREBP1),^12,14^ little is known about BCAT2 posttranscriptional regulation.

In this study, we discovered that BCAT2 is acetylated at K44. CBP and SIRT4 bind to BCAT2 and control K44 acetylation in response to BCAA availability. K44 acetylation of BCAT2 promotes its degradation through the ubiquitin–proteasome pathway, leading to decreased BCAA catabolism. BCAT2 acetylation suppresses BCAA catabolism and pancreatic tumor growth. Taken together, the data from our study reveal a previously unknown regulatory mechanism of BCAT2 in PDAC and provide a potential new therapeutic target for PDAC treatment.

## Results

### BCAT2 is acetylated mainly at lysine 44

Previous mass spectrometry data indicated that BCAT2 is a potential acetylated protein.^15,16^ To confirm this modification, we transfected Flag-BCAT2 into HEK293T cells and detected the acetylation level of BCAT2 by western blotting using a pan-specific anti-acetylated lysine antibody. The results showed that BCAT2 was indeed acetylated, and its acetylation level was increased ~2.1-fold after treatment with nicotinamide (NAM), an inhibitor of the sirtuin (SIRT) family of deacetylases, and trichostatin A (TSA), an inhibitor of histone deacetylases (HDACs) I, II, and IV (Fig. 1b). Furthermore, we found that BCAT2 acetylation was mainly increased in a time- and dose-dependent manner after NAM treatment, but not TSA (Fig. 1c; Supplementary Fig. S1a, b). According to previously obtained mass spectrometry data, the BCAT2 protein has three putative acetylation lysine (K) residues: K44, K321, and K374 (Supplementary Fig. S1c). To determine which lysine residue(s) is the major site(s), we mutated each of the three lysine residues individually to arginine (R). Arginine has a positive charge and is often used as a deacetylation mimetic mutant. The mutation of K44, but not the other lysine residues, resulted in a significant reduction in BCAT2 acetylation (Fig. 1d; Supplementary Fig. S1d). The major acetylation site K44 is evolutionarily conserved and is located in the N-terminus of BCAT2 (Fig. 1e).

To confirm the acetylation of endogenous BCAT2, we generated a K44 site-specific acetylation antibody (K44Ac) using a K44-acetylated peptide. A dot blot assay showed that the K44Ac antibody preferentially detected the acetylated peptide (Fig. 1f). In addition, a peptide-blocking experiment showed that the K44-acetylated peptide interacted with the K44Ac antibody, and the K44Ac signal was significantly decreased (Fig. 1g). More importantly, we detected increased K44 acetylation in the SW1990 and PANC-1 cells after NAM treatment (Fig. 1h). These data demonstrate that K44 is the major acetylation site in BCAT2 under the tested conditions.

### Acetylation promotes BCAT2 degradation via ubiquitylation without affecting its enzyme activity

**Fig. 2:**
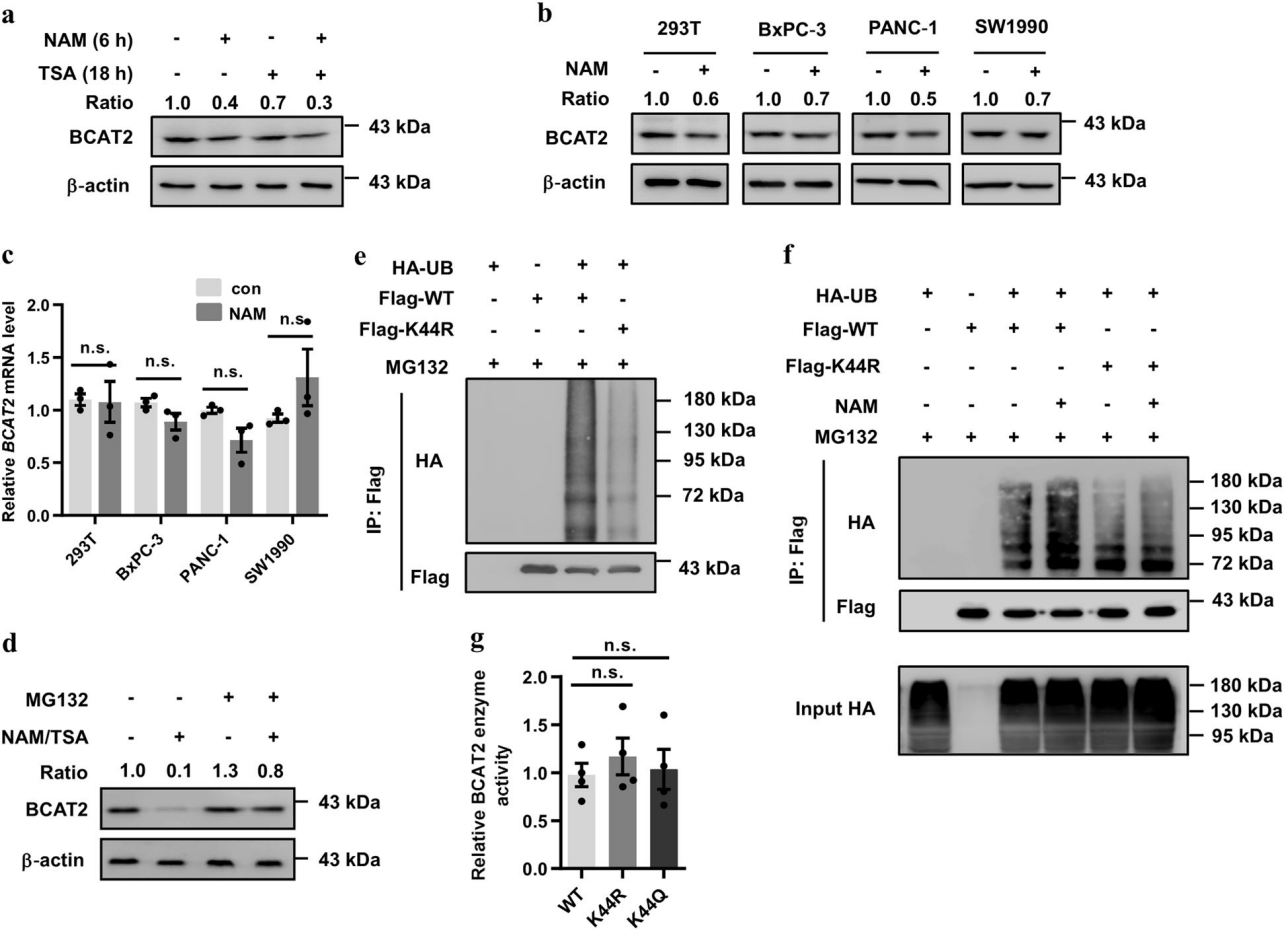
Acetylation promotes BCAT2 degradation without affecting its enzymatic activity. **a** NAM treatment decreases BCAT2 protein levels. HEK293T cells were treated with NAM, TSA, or both for the indicated times. Cell lysates were analyzed by western blotting. The relative BCAT2 protein level was normalized to that of β-actin. **b** NAM decreases BCAT2 protein in HEK293T and different PDAC cell lines. HEK293T, BxPC-3, PANC-1, and SW1990 cells were treated with NAM for the indicated times. Cell lysates were analyzed by western blotting. The relative BCAT2 protein level was normalized to that of β-actin. **c** NAM has a negligible effect on *BCAT2* mRNA levels. HEK293T, BxPC-3, PANC-1, and SW1990 cells were treated with NAM for the indicated times. The relative *BCAT2* mRNA levels were determined by qPCR and normalized to the level of *ACTB*. Results are expressed as the means ± SEM of three independent experiments. **d** MG132 blocks NAM/TSA-induced BCAT2 degradation. HEK293T cells were treated with or without NAM/TSA in the presence or absence of MG132 (10 μM, 6 h). The relative BCAT2 protein level was normalized to that of β-actin. **e** The K44R mutant has lower BCAT2 ubiquitylation levels. Flag-BCAT2 WT and the K44R mutant were coexpressed with HA-UB in HEK293T cells, and ubiquitylation of the purified protein was determined. **f** NAM increases Flag-BCAT2 WT ubiquitylation, but not that of the K44R mutant. Flag-BCAT2 WT and the K44R mutant were co-transfected with HA-UB into HEK293T cells with or without NAM treatment for the indicated time, and the ubiquitylation of the purified protein was determined. **g** Flag-BCAT2 WT activity is not significantly different from that of the K44 mutants. Flag-BCAT2 WT and the K44 mutants were expressed in HEK293T cells. The activity level of the immunopurified BCAT2 was determined. Results are expressed as the means ± SEM of four independent experiments. Significance was assessed by Student’s *t* test (**c**, **g**). n.s. no significance. Data are representative of three independent experiments in **a**, **b**, **c**, **d**, **e**, and **f**

To determine whether BCAT2 acetylation affects its protein level, we treated HEK293T cells with NAM, TSA, or both and found that BCAT2 protein levels were significantly decreased (Fig. 2a). We also found a similar phenomenon in different pancreatic cancer cells upon NAM treatment (Fig. 2b). However, *BCAT2* mRNA levels were not significantly changed after NAM treatment, indicating that this regulation was mostly achieved at the posttranscriptional level (Fig. 2c). Indeed, BCAT2 protein was rescued in HEK293T cells treated with the proteasome inhibitor MG132 (Fig. 2d), indicating that the protein stability of BCAT2 was regulated by acetylation via the ubiquitin–proteasome pathway. More importantly, the K44R mutant dramatically reduced BCAT2 ubiquitylation (Fig. 2e). Moreover, the inhibition of deacetylases by NAM increased the ubiquitylation of Flag-BCAT2 WT, but not the K44R mutant (Fig. 2f). Previous studies revealed that acetylation regulated the activity of metabolic enzymes and even acted as a switch for enzyme activity.^17–19^ Since BCAT2 is the first enzyme activated during BCAA catabolism, we measured the activity of BCAT2 WT and mutants and found no significant difference between the WT and mutants (Supplementary Fig. S2; Fig. 2g). Collectively, these results suggest that acetylation promotes BCAT2 degradation via the ubiquitin–proteasome pathway, but does not affect its activity.

### BCAA deprivation promotes BCAT2 acetylation and degradation

**Fig. 3:**
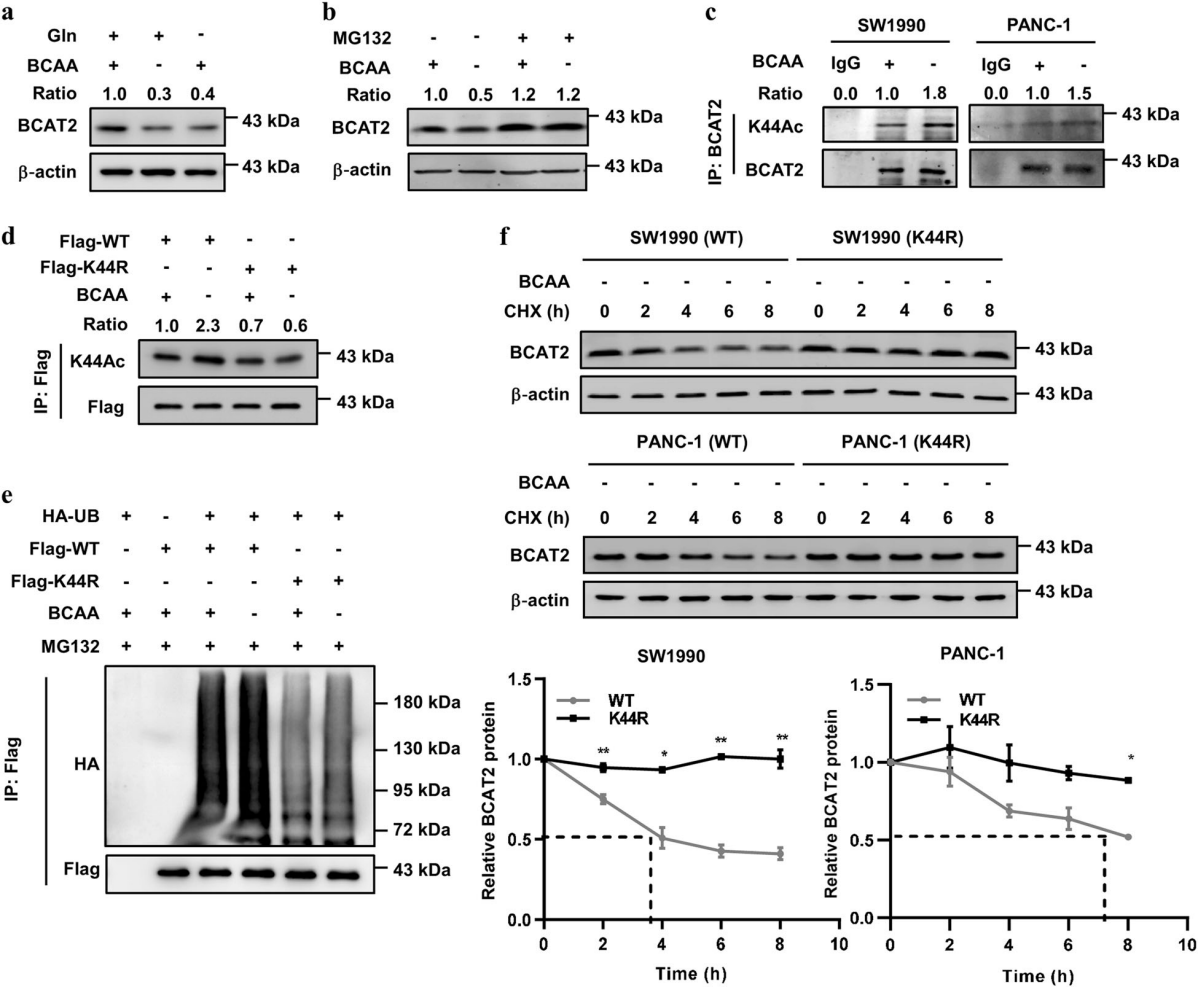
BCAA deprivation promotes BCAT2 acetylation and degradation. **a** BCAA and glutamine deprivation decreases BCAT2 protein levels. SW1990 cells were maintained in BCAA or glutamine-free medium for 24 h. Cell lysates were analyzed by western blotting. The relative BCAT2 protein level was normalized to that of β-actin. **b** MG132 blocks BCAA deprivation-induced BCAT2 degradation. SW1990 cells were maintained in BCAA-free medium for 24 h, and treated with or without MG132 (10 μM, 6 h). Cell lysates were analyzed by western blotting. The relative BCAT2 protein level was normalized to that of β-actin. **c** BCAA deprivation increases endogenous BCAT2 acetylation. SW1990 and PANC-1 cells were maintained in BCAA-free medium for 24 h, and treated with MG132 for 6 h. Cell lysates were analyzed by western blotting. The relative K44Ac acetylation level was normalized to that of BCAT2. **d** BCAA deprivation increases Flag-BCAT2 WT acetylation, but not that of the K44R mutant. Flag-BCAT2 WT and the K44R mutant were expressed in HEK293T cells and treated with NAM. BCAT2 acetylation was detected with K44Ac antibody. The relative K44Ac acetylation level was normalized to that of Flag-BCAT2. **e** BCAA deprivation increases Flag-BCAT2 WT ubiquitylation but not that of the K44R mutant. Flag-BCAT2 WT and the K44R mutant were co-transfected with HA-UB into HEK293T cells. Cells were maintained in BCAA-free medium for 24 h, and the ubiquitylation of the purified protein was determined. **f** The K44R mutant is more stable than Flag-BCAT2 WT under BCAA deprivation. Stable Flag-BCAT2 and K44R mutant cells were maintained in BCAA-free medium for 24 h, and treated with CHX (10 μg/mL) for the indicated time course. BCAT2 protein was analyzed by western blotting. Quantification of the BCAT2 protein levels is shown. The results are expressed as the means ± SEM of three independent experiments. Significance was assessed by Student’s *t* test. *P < 0.05, **P < 0.01. Data are representative of three independent experiments in **a**, **b**, **c**, **d**, **e**, and **f**

The amine groups of BCAAs can be transferred to α-ketoglutarate to produce glutamate, which can be further metabolized to glutamine, which is an energy source.^20^ To study the effect of BCAAs on the posttranslational regulation of BCAT2, we cultured cells with or without BCAAs or Gln and found that the BCAT2 protein level was obviously decreased in the SW1990 and PANC-1 cells, while MG132 blocked this effect (Fig. 3a, b; Supplementary Fig. S3), indicating that BCAT2 is regulated by proteasome-mediated degradation. To study whether BCAT2 acetylation was response to BCAA deprivation, we cultured SW1990 and PANC-1 cells with or without BCAAs in the medium and found that the endogenous K44 acetylation of BCAT2 was significantly increased (Fig. 3c). Moreover, BCAA deprivation increased Flag-BCAT2 WT, but not K44R mutant acetylation levels (Fig. 3d). We next investigated whether ubiquitylation of BCAT2 was regulated by BCAA deprivation and found that BCAA deprivation enhanced Flag-BCAT2 WT, but not K44R mutant ubiquitylation (Fig. 3e). More importantly, a cycloheximide (CHX) chase experiment indicated that the BCAT2 K44R mutant was more stable than Flag-BCAT2 WT under the tested conditions (Fig. 3f). From these results, we conclude that BCAA deprivation increases BCAT2 acetylation to promote its degradation.

### CBP acetylates BCAT2 to promote its degradation

**Fig. 4:**
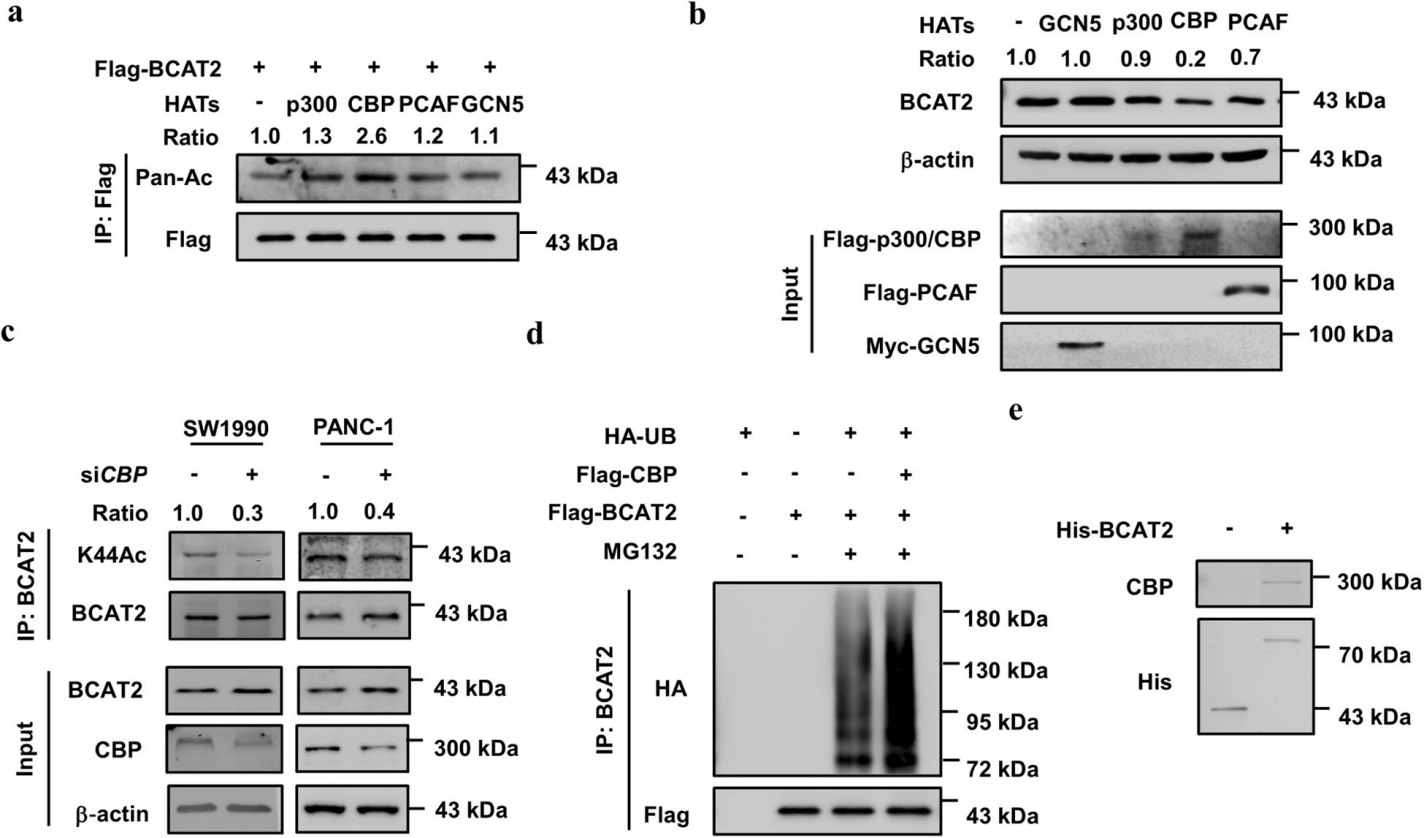
CBP acetylates BCAT2 to promote its degradation. **a** Overexpression of CBP increases BCAT2 acetylation levels. Flag-BCAT2 was expressed in HEK293T cells in combination with Flag-PCAF, Flag-P300, Flag-CBP, and Myc-GCN5. The relative BCAT2 acetylation level was normalized to that of the Flag-BCAT2 protein. **b** Overexpression of CBP decreases endogenous BCAT2 levels. Flag-PCAF, Flag-P300, Flag-CBP, and Myc-GCN5 were ectopically expressed in HEK293T cells. Endogenous BCAT2 was determined by western blotting. The relative BCAT2 protein level was normalized to that of β-actin. **c** *CBP* knockdown decreases endogenous BCAT2 acetylation. SW1990 and PANC-1 cells were transfected with si*CBP* or siNC. Endogenous BCAT2 protein was purified and detected with K44Ac antibody. The relative BCAT2 K44 acetylation level was normalized to that of the BCAT2 protein. **d** Overexpression of CBP increases BCAT2 ubiquitylation. Flag-BCAT2 was ectopically expressed in HEK293T cells in combination with Flag-CBP and HA-UB. The ubiquitylation of the purified protein was determined. **e** His-BCAT2 pulls down CBP. BL21 *E. coli* transformed with the pGEX-His-BCAT2 plasmid was induced (or not induced) by isopropyl-b-D-thiogalactoside. Protein was then purified with Ni-NTA agarose beads and incubated with SW1990 cell lysates before repurification with immunoprecipitation and subjected to western blotting. Data are representative of three independent experiments in **a**, **b**, **c**, **d**, and **e**

To identify the acetyltransferase critical for BCAT2 K44 acetylation, we transfected p300 (E1A-binding protein p300), CBP (CREB-binding protein), PCAF (KAT2B), and GCN5 (KAT2A) individually into HEK293T cells and found that CBP overexpression significantly increased the K44 acetylation level, whereas the other proteins had little effect (Fig. 4a). In addition, CBP overexpression significantly decreased the BCAT2 protein levels (Fig. 4b). Furthermore, *CBP* knockdown dramatically decreased K44 acetylation of BCAT2 and inversely increased its protein levels (Fig. 4c). In addition, overexpression of CBP increased BCAT2 ubiquitylation (Fig. 4d). The direct interaction between BCAT2 and CBP was verified by a His-BCAT2 pull-down assay (Fig. 4e). Collectively, these data indicate that CBP acetylates BCAT2 at K44 to promote its degradation.

### BCAT2 is stabilized by SIRT4 deacetylation

**Fig. 5:**
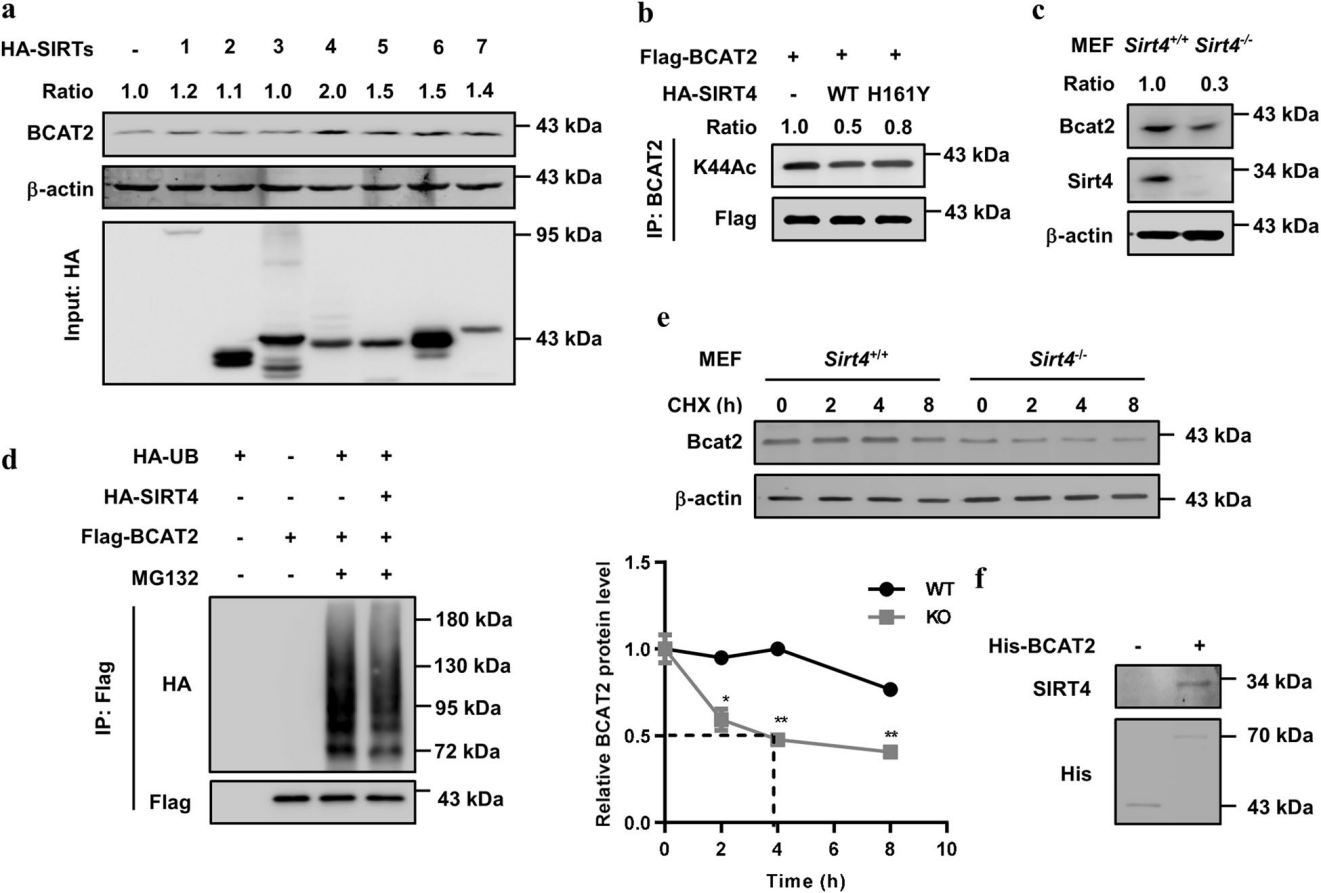
BCAT2 is stabilized by SIRT4 deacetylation. **a** SIRT4 overexpression increases endogenous BCAT2 protein levels. HA-SIRT1-7 was expressed in HEK293T cells. Endogenous BCAT2 was determined by western blotting. The relative BCAT2 protein level was normalized to that of β-actin. **b** SIRT4 deacetylates BCAT2. Flag-BCAT2 was ectopically expressed in HEK293T cells in combination with SIRT4 WT and H161Y mutant. The relative BCAT2 acetylation level was normalized to that of Flag-BCAT2. **c** Bcat2 is decreased in *Sirt4-*knockout (KO) MEFs. Bcat2 protein was determined by western blotting. Relative Bcat2 protein level was normalized to β-actin. **d** Overexpression of SIRT4 decreases BCAT2 ubiquitylation. Flag-BCAT2 was ectopically expressed in HEK293T cells in combination with SIRT4 WT and HA-UB. The ubiquitylation of purified protein was determined. **e** *Sirt4* KO destabilizes Bcat2 in MEFs. *Sirt4* WT and KO MEFs were subjected to CHX treatment (10 μg/mL) for the indicated time course. Bcat2 protein was analyzed by western blotting. Quantified Bcat2 protein levels are shown. The results are expressed as the mean ± SEM of three independent experiments. The significance was assessed by Student’s *t* test. *P < 0.05, **P < 0.05. **f** His-BCAT2 readily pulls down SIRT4. BL21 *E. coli* transformed with the pGEX-His-BCAT2 plasmid was induced (or not induced) by isopropyl-b-D-thiogalactoside. Protein was then purified through a Ni-NTA column and incubated with SW1990 cell lysates before repurification with immunoprecipitation and subjected to western blotting. Data are representative of three independent experiments in **a**, **b**, **c**, **d**, **e**, and **f**

According to the results that NAM increases BCAT2 acetylation, we speculated that SIRT family members might deacetylate BCAT2. To identify which deacetylase is the major deacetylase, we overexpressed SIRT1–7 individually with BCAT2 in HEK293T cells and found that SIRT3 and SIRT4 readily bound to BCAT2 (Supplementary Fig. S4). Notably, we also found that SIRT4, but not SIRT3, increased BCAT2 protein levels (Fig. 5a), indicating that SIRT4 is the potential deacetylase critical for BCAT2 deacetylation. Consistently, SIRT4 WT but not the catalytically inactive mutant H161Y^21^ decreased exogenous BCAT2 acetylation in HEK293T cells (Fig. 5b). Interestingly, we found that the expression of Bcat2 was lower in embryonic fibroblasts (MEFs) from *Sirt4*-knockout mice than it was in WT mice (Fig. 5c). Furthermore, overexpression of SIRT4 decreased the ubiquitylation of BCAT2 (Fig. 5d). CHX chase experiments showed that endogenous Bcat2 in *Sirt4*-knockout MEFs had a dramatically decreased half-life (Fig. 5e). The direct interaction between BCAT2 and SIRT4 was verified by a His-BCAT2 pull-down assay (Fig. 5f). Taken together, these findings indicate that SIRT4 is a deacetylase for BCAT2 K44 acetylation under the tested conditions.

### K44 mutants promote pancreatic cancer growth

**Fig. 6:**
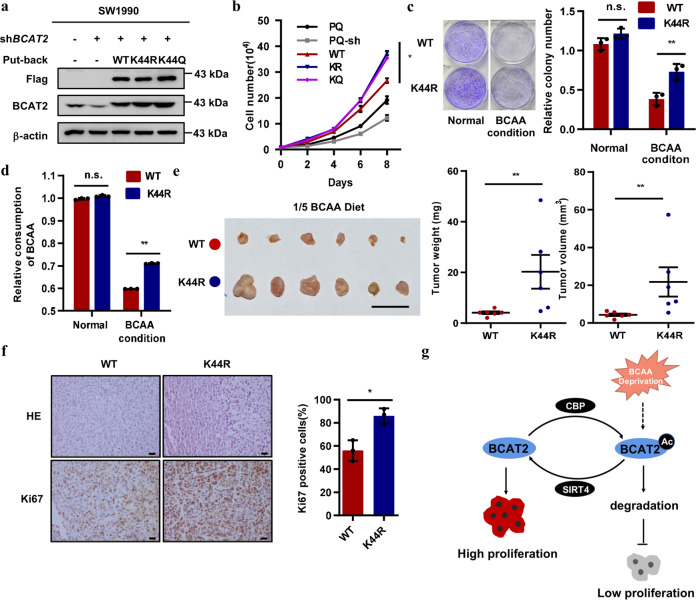
The K44R mutant promotes pancreatic cancer growth. **a** Verification of the stable SW1990 cell lines. *BCAT2* was stably knocked down with shRNA in SW1990 cells. shRNA-resistant Flag-BCAT2 WT and K44R/Q mutants were stably re-expressed, and the knockdown efficiency and re-expression levels were determined by western blotting. **b** The K44 mutants promote cell proliferation. Cells were seeded in 12-well plates in BCAA-free medium. Cells were counted every 48 h. Error bars represent cell number ± SEM for experiments performed in triplicate. **c** The K44R mutant promotes colony formation. The cells were seeded in six-well plates and maintained in BCAA-free medium or normal medium. The results are expressed as the means ± S.D. of three independent experiments. **d** The K44R mutant has increased BCAA uptake. The cells were pretreated with BCAA-free medium or normal medium for 24 h. Then, after the cells were grown for 24 h, the BCAA concentration in the culture medium was measured. The results are expressed as the means ± S.D. of three independent experiments. **e** The K44R mutant promotes tumor growth in vivo. A subcutaneous xenograft experiment was performed with nude mice using *BCAT2*-knockdown SW1990 cells re-expressing Flag-BCAT2 WT and the K44R mutant. Xenograft tumors were dissected, and their weight and volume were determined. WT: mean ± SEM of *n* = 6; K44R: mean ± SEM of *n* = 6. **f** The K44R mutant in the tumor xenograft samples has increased Ki67 staining. Cells were quantified and analyzed (right). The percentage of Ki67-positive cells is presented as the mean ± S.D. calculated from three different views. Significance was assessed by Student’s *t* test (**c**–**e**, **f**) and one-way ANOVA (**b**). *P < 0.05, **P < 0.01, n.s. no significance. Scale bar, 50 µm. Data are representative of three independent experiments as shown in **a**, **c**, **d**. **g** Working model depicting how acetylation at K44 promotes BCAT2 degradation in response to BCAA deprivation. This downregulated expression of BCAT2 contributes to the suppression of BCAA catabolism and tumor cell proliferation

To investigate whether BCAT2 acetylation is important for pancreatic cancer cells, we examined the effect of BCAT2 acetylation on pancreatic cancer cells. We knocked down endogenous *BCAT2* using short-hairpin RNA at the 3′-UTR and stably re-expressed WT and K44-mutant BCAT2 in SW1990 cells (Fig. 6a). We performed a cell count assay to evaluate the proliferation of stable Flag-BCAT2 WT and K44-mutant cells under normal and BCAA-deprived conditions. Stable SW1990 cells expressing either the K44R or K44Q mutant proliferated significantly faster than did the cells expressing WT under BCAA-deprived conditions (Fig. 6b). However, there was no significant difference between stable WT and mutant cell proliferation under normal conditions (data not shown). The colony-formation assay showed that the K44R mutant produced more clones than did the WT cells under BCAA-deprived conditions (Fig. 6c). In addition, the stable K44R mutant cells showed increased BCAA uptake after pretreatment under BCAA deprivation conditions (Fig. 6d), and the BCAA levels in the Flag-BCAT2 WT cells, but not the stable K44R mutant cells, were significantly decreased (Supplementary Fig. S5a). To also explore the effect of BCAT2 acetylation on pancreatic cancer cell proliferation in vivo, we performed xenograft experiments using stable BCAT2 WT and K44R mutant cells. The results showed that the stable cells expressing the K44R mutant induced significantly faster tumor growth than did the stable WT cells treated with 1/5 BCAA, while the effect under normal conditions was less pronounced, it was still significant (Fig. 6e; Supplementary Fig. S5b). In addition, we found Ki67 staining to be more intense in the tumor xenograft cells re-expressing BCAT2 K44R than it was in the Flag-BCAT2 WT cells (Fig. 6f). Collectively, these data indicate that K44 mutants stabilize BCAT2 and promote pancreatic tumor cell growth upon BCAA deprivation both in vitro and in vivo.

## Discussion

Lysine acetylation was first discovered on histone tail lysine residues,^22^ and then nonhistone proteins were also found to be modified by acetylation.^23^ Many mitochondrial proteins are modified by acetylation, and over 60% of mitochondrial proteins contain acetylation sites.^24^ BCAT2 is reported to be located in the mitochondria and an important enzyme of BCAA catabolism. In this study, we demonstrate that BCAT2 is regulated by acetylation in response to BCAA deprivation. CBP and SIRT4 control the K44 acetylation level. K44 acetylation of BCAT2 promotes its degradation through the ubiquitin–proteasome pathway, leading to decreased BCAA catabolism. BCAT2 acetylation suppresses BCAA catabolism and PDAC cell proliferation (Fig. 6g).

Acetylation is a dynamically reversible regulatory process. We have identified SIRT4 as a deacetylase responsible for BCAT2 acetylation. SIRT3, SIRT4, and SIRT5 are all located in the mitochondria. However, they have different functions. SIRT3 has strong deacetylase activity,^25^ and SIRT5 has demalonylase, desuccinylase, and deglutarylase activity.^26–28^ Although SIRT4 is reported to have a weak deacetylase, some substrates have been found to be deacetylated by it.^29^ An elegant study found that SIRT4 directly bound, deacetylated, and inhibited malonyl-CoA decarboxylase (MCD).^30^

In our study, we uncovered the importance of acetylation in regulating stable BCAT2 levels and found that Flag-BCAT2 WT was more easily degraded than the K44R mutant in response to BCAA deprivation. However, this finding led to another question: does K44 acetylation influence BCAT2 activity? We found that the acetylation of BCAT2 had no effect on its activity. This outcome may be due to the K44 acetylation site being located in the N-terminus, far from the enzyme active center.

On the basis of the data showing that BCAA deprivation promotes BCAT2 degradation in vitro, we used the 1/5 BCAA to manipulate the BCAA-deprivation condition and observed the effect of K44 acetylation on BCAT2-induced pancreatic tumor cell growth in vivo. The 1/5 BCAA treatment was also used for nude mice. Because the BCAAs could be directly incorporated into proteins, the treated nude mice had low body weight. A study reported that BCAAs acted as signaling molecules, especially leucine, and may activate the mTOR pathway to support cell growth.^31^

To check the correlation between BCAT2 and its acetyltransferase or deacetylase, we analyzed the correlation between CBP and BCAT2 expression in pancreatic cancer patients in a TCGA cohort and found that BCAT2 was negatively correlated with CBP (Supplementary Fig. S6a). Similarly, we found that BCAT2 was positively correlated with SIRT4 in the TCGA cohort (Supplementary Fig. S6b). However, these correlations were weak at the mRNA level, and further study is needed.

Recently, BCAA metabolism in many cancers has attracted substantial attention.^9,11–13,32^ However, BCAA metabolism plays various functional roles in different cancer types, which may be determined by both tissue-of-origin and oncogenic mutations.^33^ The KRAS oncogene is a driver mutation, and ~90% of pancreatic ductal adenocarcinomas have KRAS mutations. In contrast to that in pancreatic cancer, the mutation rate of KRAS in liver cancer is very low. Interestingly, a recent article reported that BCAA catabolism was lost during liver cancer development and progression.^32^ This study highlighted that accumulated BCAAs were used as signal molecules, and that dietary BCAA interventions could effectively modulate HCC development and growth. Similarly, we observed that the tumors were smaller in nude mice fed with 1/5 the amount of BCAA than they were in mice fed with a normal diet. Dietary interventions may be good treatment and prevention strategies.

Pancreatic ductal adenocarcinoma has a poor prognosis, and is projected to be the second leading cause of cancer death by 2030.^34^ Abnormal branched-chain amino acid metabolism occurs at the early stage of pancreatic cancer.^7^ Our study showed that BCAA deprivation induces BCAT2 degradation to suppress BCAA catabolism and pancreatic cancer growth. We also revealed a newly discovered regulatory mechanism of BCAT2 that controls its stability. Based on the posttranslational regulation of BCAT2, targeting BCAT2 may be a strategy for the prevention and treatment of PDAC.

## Materials and methods

### Plasmid construction

The full-length cDNA of *BCAT2* was cloned into the indicated vectors. Point mutations of *BCAT2* were generated by site-directed mutagenesis. *BCAT2* shRNA oligos were synthesized, annealed, and cloned into the pMKO.1 vector. All expression constructs were verified by DNA sequencing.

### Antibodies

Antibodies against the following proteins were used: β-actin (#9601, Aogma, USA), Flag (#9622, Aogma), HA (#T501, Signalway Antibody, College Park, MD, USA), HIS (#12698, Cell Signaling Technology, Danvers, MA, USA), BCAT2 (#9432, Cell Signaling Technology), SIRT4 (#HPA029691, Sigma-Aldrich, Merck KGaA, Darmstadt, Germany), CBP (#2832, Abcam, Cambridge, MA, USA), and Ki67 (#15580, Abcam). Pan-acetyl lysine polyclonal antibodies were generated using chemically modified acetylated chicken ovalbumin as an antigen. To generate a site-specific antibody to detect the acetylation of K44 in BCAT2, the synthesized acetylated peptide (LEMTQK (Ac) PHKKPGP) was used as an antigen to immunize rabbits. Antiserum was collected after five doses of administered for the immunization.

### Cell culture and treatment

Human pancreatic carcinoma cell lines (SW1990, PANC-1, BxPC-3) and HEK293T cells were obtained from the American Type Culture Collection (Manassas, VA, USA) and cultured in Dulbecco’s modified Eagle’s medium (DMEM) supplemented with 10% fetal bovine serum (Gibco, Thermo Fisher Scientific, Waltham, MA, USA) in the presence of 100 units/mL penicillin and 100 μg/mL streptomycin (Gibco, Thermo Fisher Scientific). The cells were grown in a cell culture incubator at 37 °C and 5% CO_2_. The treatment of NAM/TSA, MG132, and CHX is described in the figure legends.

### siRNA transfection and RNA interference

CBP was downregulated by RNA interference. Synthetic siRNA oligonucleotides were obtained commercially from Shanghai GenePharma Co, Ltd. The following effective sequences were used:

si*CBP*-1: 5′-GGAAGCAGCUGUGUACCAUTTdTdT-3′

si*CBP*-2: 5′-GCAUGAAUGCUAACUUUAAdTdT-3′

si*CBP*-3: 5′-CCUACAGAUAUCAAGAAUAdTdT-3′

The transfection of each siRNA was performed with Lipofectamine RNAiMAX (Invitrogen, Thermo Fisher Scientific) following the manufacturer’s instructions. The knockdown efficiency was verified by western blotting.

### Immunoprecipitation and western blotting

Cells were lysed in 0.3% Nonidet P40 buffer (150 mM NaCl and 50 mM Tris-HCl, pH 7.5) containing protease inhibitor cocktail (Selleck, Houston, TX, USA), TSA (30 μM) and NAM (15 mM). The cell lysates were incubated with Flag beads at 4 °C for 3 h after cell debris was removed by centrifugation at 16,200 *g* for 15 min and 4 °C. After incubation, the samples were washed with lysis buffer three times. Then, the beads were boiled in 1× SDS loading buffer and centrifuged at 4 °C in preparation for the western blot analysis.

### Ubiquitin ladder assay

A ubiquitin ladder assay was performed as previously described.^35^ Cells were collected 48 h after transfection and lysed in 1% SDS buffer (50 mM Tris-HCl, pH 7.5; 0.5 mM EDTA; and 1 mM DTT) containing protease inhibitors and boiled for 10 min. The cell lysates were diluted ten-fold with 0.3% Nonidet P40 buffer for immunoprecipitation. Ubiquitylation was determined by western blotting.

### RNA isolation and qRT-PCR

The total RNA was extracted from SW1990, PANC-1, BxPC-3, and HEK293T cells using TRIzol and reverse transcribed to cDNA. Real-time PCR was performed using SYBR Premix Ex Taq (#RR420A, TaKaRa Bio, Kusatsu, Japan). The relative gene expression was calculated by the comparative CT method using *ACTB* as a control. The following primer sequences were used:

h*ACTB* forward primer: 5′-GCACAGAGCCTCGCCTT-3′

h*ACTB* reverse primer: 5′-GTTGTCGACGACGAGCG-3′

h*BCAT2* forward primer: 5′-GCTGGTCTTTGCCTTTGAAC-3′

h*BCAT2* reverse primer: 5′-CCTTCCAGAACCTCACGCT-3′.

### His pull-down assay

BL21 *E. coli* transformation with the pGEX-His-BCAT2 plasmid was induced with isopropyl-b-D-thiogalactoside (0.1 mM as final concentration) at 16 °C for 12 h. The his-BCAT2 protein was purified through nickel-nitrilotriacetic acid (Ni-NTA) agarose beads (#P6611, Sigma-Aldrich), added to HEK293T cell lysate, and incubated overnight at 4 °C. The beads were harvested by centrifugation and washed three times with 0.3% NP-40 buffer. Then, the samples were boiled in 1× SDS loading buffer and subjected to western blot analysis.

### Determination of BCAT2 activity

BCAT2 activity was assessed as described previously.^36^ To assay BCAT2 enzymatic activity, reactions were conducted in 96-well plates in 200 μL of 100 mM potassium phosphate buffer (pH 7.4) containing 5 μM PLP, 50 mM ammonium sulfate (100 mM NH_4_^+^), 0.05 mM NADH, 5 mM DTT, 5 mM a-ketoglutarate, 10 mM L-leucine, and 25 μg (0.95 U) of LeuDH. The mixture was preincubated at 37 °C, and then, Flag-BCAT2 and Flag-BCAT2 mutants were added separately to the reaction mixture to initiate the reaction. The disappearance of NADH absorbance at 340 nm was monitored continuously in a spectrofluorometer (FL-4600, Hitachi). Triplicate-independent experiments were performed.

### Construction of stable cell pools and cell proliferation analysis

Flag-tagged human wild-type (WT), K44R mutant, and K44Q mutant BCAT2 were cloned into a retroviral vector (pQCXIH) and co-transfected with vectors expressing *gag* and *vsvg* genes into HEK293T cells. The retroviral supernatants were harvested and applied to SW1990 and PANC-1 cells. The SW1990 and PANC-1 cells were infected with the prepared virus for 48 h, and screened by hygromycin for at least 2 weeks. pMKO-sh*BCAT2* and pMKO-shVEC were constructed as short-hairpin RNA vectors. Stable SW1990 cells re-expressing WT, K44R, and K44Q BCAT2 were infected with pMKO-sh*BCAT2* and pMKO-shVEC viruses, and the *BCAT2-*knockdown efficiency was determined after selection with puromycin for 2 weeks. The following shRNA targeting the *BCAT2* 3′-untranslated region was used:

5′-CCGGACTACAAGTTAGGTGGGAATTCTCGAGAATTCCCACCTAACTTGTAGTTTTTTG-3′. For the cell proliferation assay, 8 × 10^3^ SW1990 cells or PANC-1 cells were seeded in triplicate in 12-well plates. The number of cells was counted every 48 h for 8 days.

### Measurement of BCAA concentration

The BCAA concentration in the medium was measured by a branched-chain amino acid kit (MAK003, Sigma-Aldrich) according to the manufacturer’s instructions. The absorbance of the wells in triplicate 96-well plates was measured at 450 nm.

### Xenograft analysis

The procedures related to animal studies were approved by the animal care committee of Fudan University (Shanghai, China). Stable SW1990 cells re-expressing WT BCAT2 or the K44R mutant were mixed with 50% PBS and 50% Matrigel (#354248, Corning, Corning, NY, USA), and BALB/c nude mice (male, 6-weeks old) were purchased from Shanghai SIPPR-BK Laboratory Animal Company (Shanghai, China). The mice were injected subcutaneously with 1 × 10^7^ cells. The mice were subsequently fed a normal BCAA diet or a 1/5 BCAA diet. Tumors were harvested 8 weeks after injection, and the tumor volume and weight were calculated.

### Ki67 staining

Ki67 staining was performed with formalin-fixed paraffin-embedded xenograft tissues. Following deparaffinization, antigen retrieval, and incubation with goat serum for 30 min, the sections were incubated overnight with the Ki67 antibody (#15580, Abcam) at a 1:200 dilution at 4 °C and then incubated for 45 min with horseradish peroxidase (HRP)-conjugated anti-rabbit secondary antibody. Finally, diaminobenzidine (DAB) staining was performed according to the manufacturer’s instructions.

### Statistics

Statistical analysis was performed by a two-tailed unpaired Student’s *t* test or one-way ANOVA. The data shown represent the results obtained from triplicate-independent experiments (mean ± S.D. or SEM). *P* values < 0.05 were considered significant (*P < 0.05, **P < 0.01, ***P < 0.001, and n.s. no significance).

## Supplementary information

Acetylation promotes BCAT2 degradation to suppress BCAA catabolism and pancreatic cancer growth
