# Supplementary material for: Acetylation promotes BCAT2 degradation to suppress BCAA catabolism and pancreatic cancer growth
Source: Signal Transduct Target Ther. 2020 May 29;5:70. doi: 10.1038/s41392-020-0168-0 (PMC7256045; doi:10.1038/s41392-020-0168-0)
Supplement: Supplementary file 1 — Acetylation promotes BCAT2 degradation to suppress BCAA catabolism and pancreatic cancer growth [file 41392_2020_168_MOESM1_ESM.docx]

Supplementary Materials for

Acetylation promotes BCAT2 degradation to suppress BCAA catabolism and pancreatic cancer growth

Ming-Zhu Lei^1,2^, Xu-Xu Li^1,2^, Ye Zhang^1^, Jin-Tao Li^2,3^, Fan Zhang^2,3^, Yi-Ping Wang^2,3^, Miao Yin^2,3^, Jia Qu^2,3*^, Qun-Ying Lei^1,2,3,4*^

Correspondence to: qujia0207@163.com or qlei@fudan.edu.cn

**This PDF file includes:**

Figures. S1 to S6

**Figure. S1.**

**a**

**b**

**+**

**+**

**+**

**+**

**+**

**+**

**+**

**+**

**-**

**Flag-BCAT2**

**Flag-BCAT2**

**+**

**+**

**+**

**-**

**-**

**-**

**-**

**TSA**

**NAM**

**+**

**-**

**+**

**-**

**NAM 5 mM (h)**

**0**

**4**

**6**

**8**

**2**

**
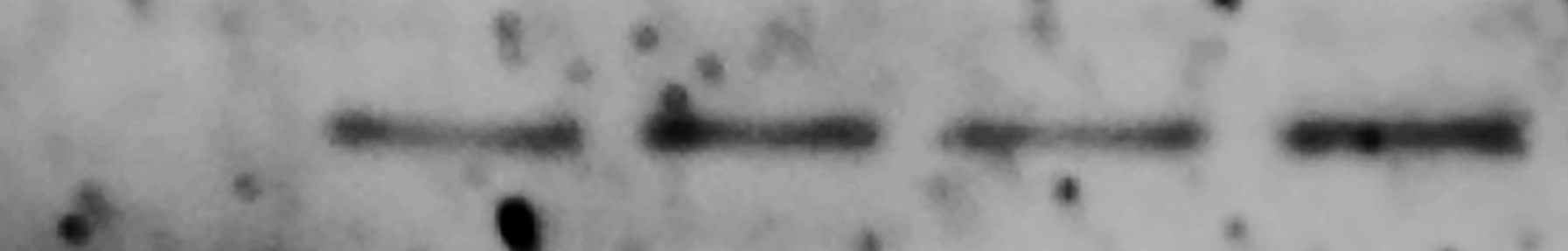

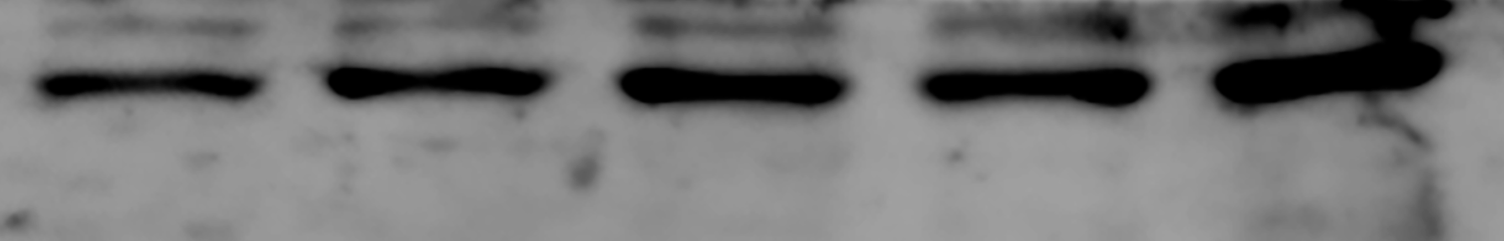
**

**1.6**

**2.5**

**1.2**

**2.0**

**\**

**1.6**

**1.3**

**1.0**

**43 kDa**

**2.0**

**Ratio**

**Pan-Ac**

**IP: Flag**

**Ratio**

**43 kDa**

**Pan-Ac**

**0.0**

**1.0**

**
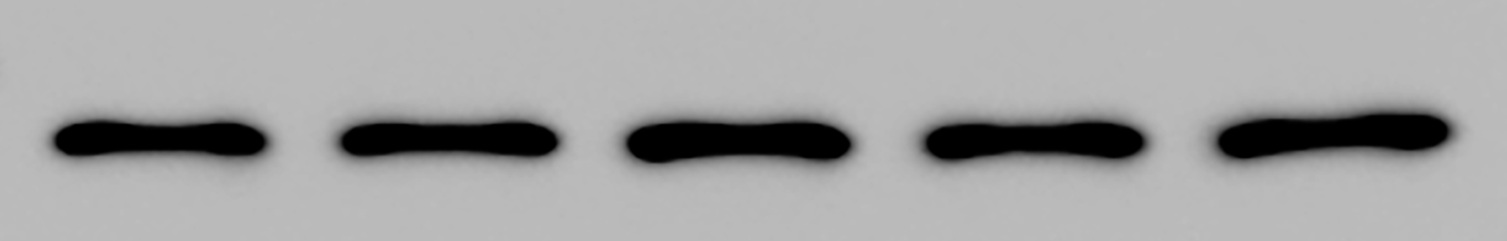

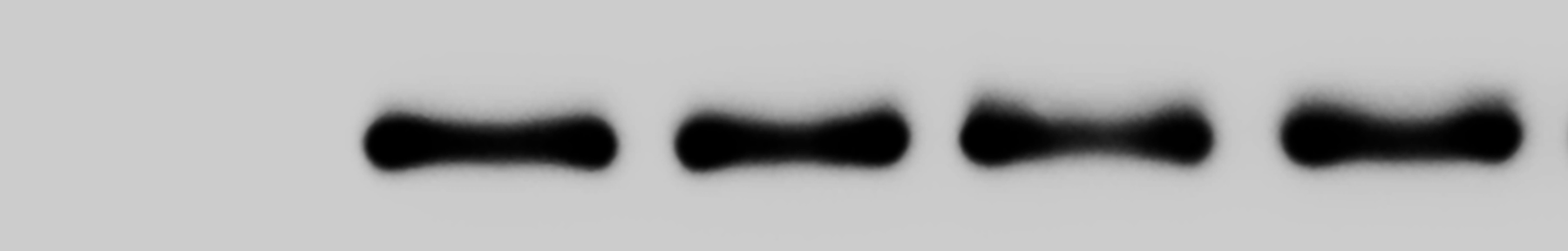
**

**43 kDa**

**IP: Flag**

**Flag**

**43 kDa**

**Flag**

**c**

**d**


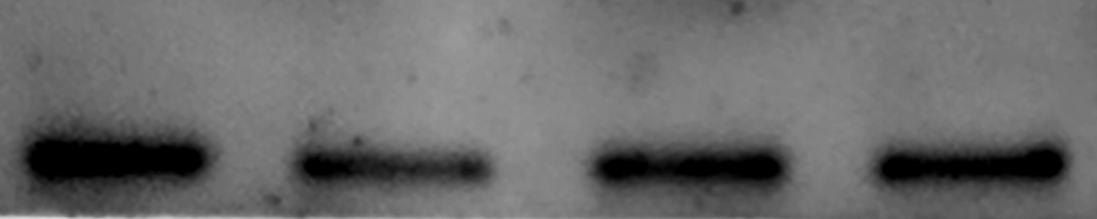

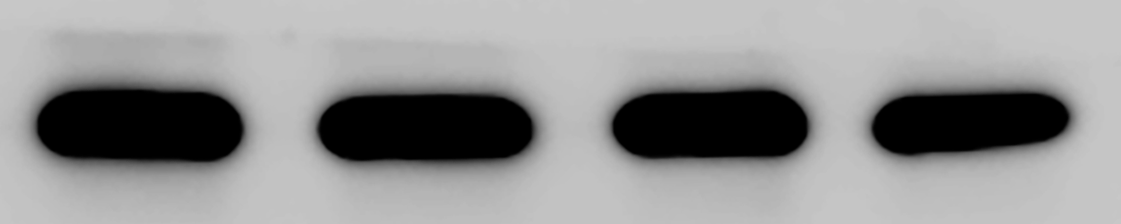


**0.4**

**0.9**

**43 kDa**

**0.9**

**1.0**

**IP: Flag**

**43 kDa**

**Flag-BCAT2**

**K44R**


**K321R**


**K374R**


**WT**

**Ratio**

**Flag**

**Pan-Ac**

| **Position** | **Mascot Score** | **PTM Score** | **Modified Sequence** |
| --- | --- | --- | --- |
| **44** | **41.50** | **139.73** | **_QLEMTQK(ac)PHKKPG_** |
| **321** | **49.67** | **132.16** | **_ERTITMK(ac)QLLRAL_** |
| **374** | **29.84** | **77.92** | **_RFQK(ac)ELKEIQYGI_** |

**Supplemental Fig. S1. Acetylation of BCAT2 is mainly at Lysine 44.** **a** NAM increases BCAT2 acetylation in a time-dependent manner. Flag-BCAT2 was transfected into HEK293T cells. Cells were treated with 5 mM NAM for the indicated time. Relative BCAT2 acetylation was normalized by Flag-BCAT2 protein. **b** NAM but not TSA increases BCAT2 acetylation. Flag-BCAT2 was transfected into HEK293T cells. Cells were treated with NAM, TSA or both for the indicated time. Relative BCAT2 acetylation was normalized by Flag-BCAT2 protein. **c** Identification of acetylated BCAT2 peptide by mass spectrometry. **d** K44R mutant decreases BCAT2 acetylation. Flag-BCAT2 WT, K44R, K321R and K374R mutants were ectopically transfected in HEK293T cells. Relative BCAT2 acetylation was normalized by Flag-BCAT2 protein. Data are representative of three independent experiments in **a**, **c** and **d**.

**Figure. S2.**


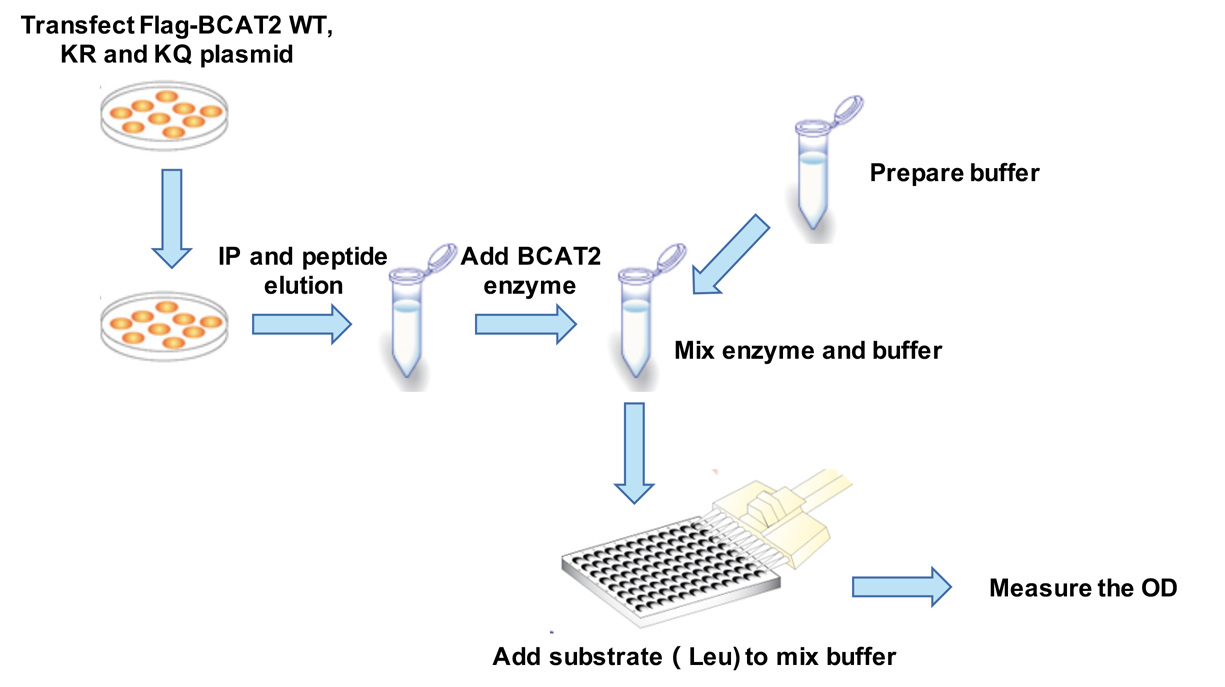


**Supplemental Fig. S2. Schematic diagram of BCAT2 activity detection.** Flag-BCAT2 WT, K44R and K44Q mutants were ectopically expressed in HEK293T cells and were immunoprecipitated with Flag-beads. And then they were eluted and separately added to the reaction mixture to initiate the reaction. The disappearance of absorbance of NADH at 340 nm was monitored continuously in a spectrofluorometer.

**Figure. S3.**

**PANC-1**

**+**

**-**

**-**

**+**

**BCAA**

**+**

**-**

**Ratio**

**0.9**

**0.5**

**1.0**

**1.0**

**-**

**+**

**MG132**

**
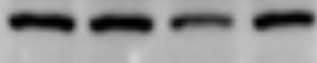
**

**43 kDa**

**BCAT2**

**
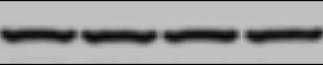
**

**43 kDa**

**β-actin**

**Supplemental Fig. S3. MG132 blocks BCAA deprivation-induced BCAT2 degradation.** PANC-1 cells were maintained in BCAA free medium for 24 h and treated with or without MG132 (10 μM, 6 h). Cell lysates were analyzed by western blotting. Relative BCAT2 protein was normalized by β-actin. Data are representative of three independent experiments.

**Figure. S4.**

**7**

**3**

**4**

**5**

**6**

**2**

**HA-SIRTs**

**-**

**1**

**+**

**+**

**+**

**+**

**+**

**+**

**+**

**+**

**Flag-BCAT2**


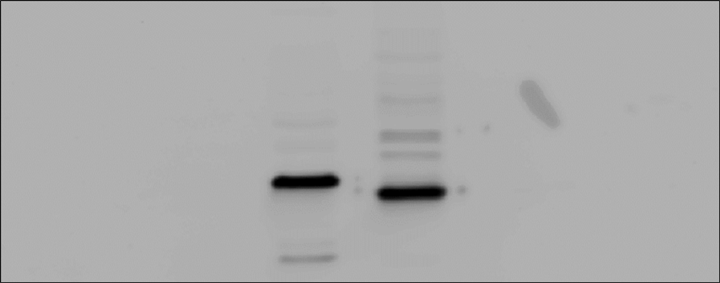


**IP: Flag**

**HA**

**43 kDa**


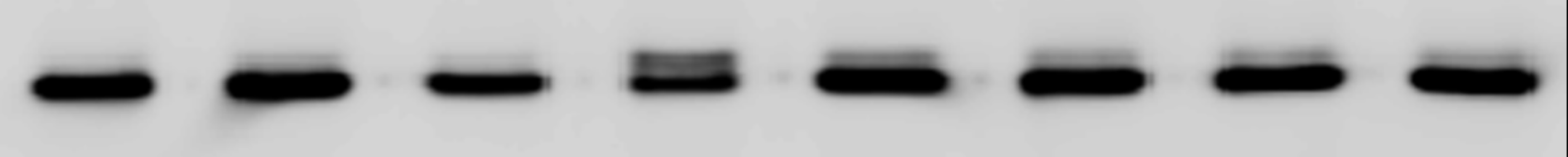


**43 kDa**

**Flag**


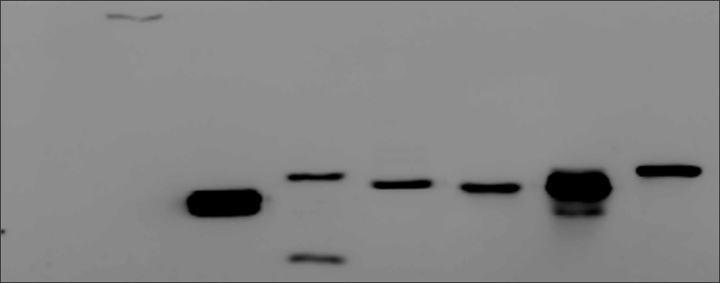


**95 kDa**

**HA**

**Input**

**43 kDa**


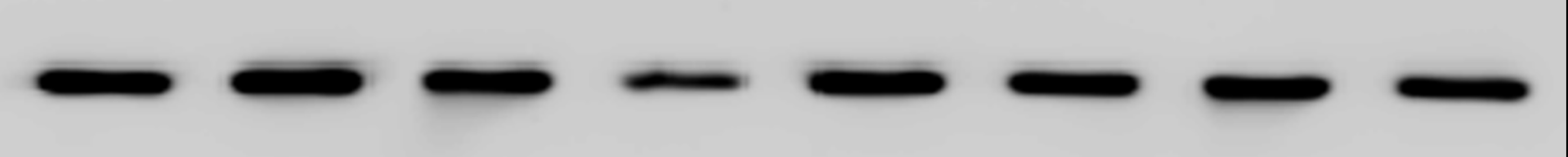


**43 kDa**

**Flag**

**Supplemental Fig. S4. BCAT2 binds to SIRT3 and SIRT4.** Flag-BCAT2 was expressed in HEK293T cells in combination with HA-tagged SIRTs (1-7). Flag-BCAT2 was immunoprecipitated with Flag-beads and analyzed by western blotting. Data are representative of three independent experiments.

**Figure. S5.**

**a**

**Normal Diet**

**WT**

**K44R**


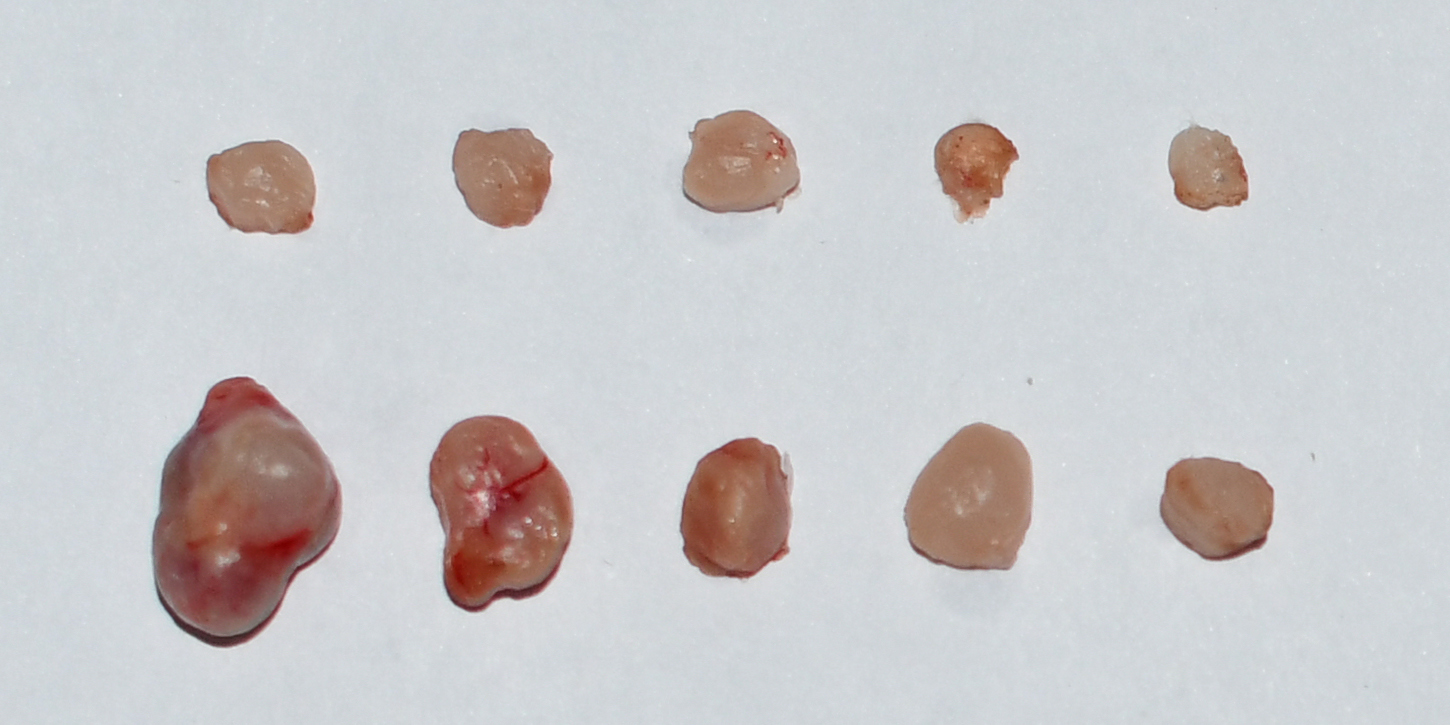


**b**

**Supplemental Fig. S5. K44R mutant promotes tumor growth *in vivo*. a** BCAA concentration of SW1990 Flag-BCAT2 WT stable cells is significantly [decrease](E:/Program%20Files/Youdao/Dict/8.8.0.0/resultui/html/index.html#/javascript:;)d under BCAA deprivation treatment. 2×10^6^ SW1990 Flag-BCAT2 and K44R mutant SW1990 stable cells were maintained in BCAA free medium for 24 h, and then BCAA levels inside the cells were measured. ****P* < 0.001. n.s., no significance. Mean ± S.D. of n = 5. **b** Subcutaneous xenograft experiment was performed in nude mice using the *BCAT2*-knockdown SW1990 cells re-expressing Flag-BCAT2 WT or K44R mutant. Xenograft tumors were dissected for weight and volume determination. **P* < 0.05. WT: mean ± SEM. of n = 5; K44R: mean ± SEM. of n = 5.

**Figure. S6.**

**b**

**a**

**Supplemental Fig. S6. BCAT2 expression levels are negatively and positively correlated with CBP and SIRT4 respectively in TCGA cohort.** **a** BCAT2 negatively correlates with CBP expression in TCGA cohort of pancreatic cancer patients. **b** BCAT2 positively correlates with SIRT4 expression in TCGA cohort of pancreatic cancer patients.
